# A Review of Anemia Prevalence, and Prevention and Control Strategies, in the Eastern Europe and Central Asia Region

**DOI:** 10.1016/j.cdnut.2024.104477

**Published:** 2024-10-15

**Authors:** Jacky Knowles, Tamsin Walters, Amirhossein Yarparvar, Rebecca Brown

**Affiliations:** 1 NutritionWorks, Bristol, United Kingdom; 2 UNICEF, Sama Beirut, Beirut, Lebanon

**Keywords:** maternal health and nutrition, young child health and nutrition, women of reproductive age, adolescent health and nutrition, nutrition specific, nutrition sensitive

## Abstract

Anemia is a global public health and nutrition problem. However, data on its prevalence and potential causal factors in the 22 countries of the UNICEF Europe and Central Asia region are not systematically collected, analyzed, and reported, leading to challenges in formulating appropriate preventative strategies. We examined available anemia prevalence data among different population groups through reviewing survey reports from 2010 to 2022; conducted a literature review to explore underlying determinants of anemia including iron deficiency; and collated and reviewed program and policy documentation across the region. Anemia prevalence data for the period 1999–2009 were also researched to examine trends in anemia prevalence in the region over the past 23 y. Nationally representative data for anemia for ≥1 population group since 2010 were found for half the 22 countries, whereas less than a quarter of countries had recent data for iron deficiency. There was a lack of evidence for other factors potentially contributing to anemia in the region. Where information was available, our findings highlight that anemia remained a problem of public health significance among girls and women 15–49 y old and preschool children in all countries with data; anemia was highly associated with iron deficiency; large discrepancies in anemia prevalence existed between geographical regions and subpopulation groups within countries; and only a few countries were implementing the recommended WHO strategies to prevent and control anemia. The paucity of recent, representative, data on anemia in many countries and on the etiology of anemia in most countries remain obstacles to ensuring that effective anemia prevention strategies are placed high on national agendas in the region.

## Introduction

Anemia is a public health and nutrition problem with serious consequences, for example increased maternal and perinatal mortality, adverse effects on cognitive development in children, and reduced physical capacity and work productivity in adults [1]. The consequences of anemia are recognized by the fact that anemia prevalence among women of reproductive age is 1 of the indicators for Sustainable Development Goal 2 (SDG Indicator 2.2.3) and that a 50% reduction in anemia in women of reproductive age from 2012 levels is 1 of the World Health Assembly 2025 global targets [2]. The etiology of anemia is recognized to be multifactorial and can include nutrition-specific and non-nutritional factors [[3], [4], [5]]. Studies that modeled the predictors of anemia among preschool children 6–59 mo of age [6] and women of reproductive age [7] respectively, found that, in general, in settings with low and moderate burdens of infectious disease, a higher proportion of anemia can be attributed to nutritional factors such as iron deficiency when compared with settings with a high infection burden.

Despite its significance for public health and national productivity, anemia is often a low priority on the agendas of ministries of health, potentially because its prevalence and causes are unknown or under-estimated, especially in middle- to high-income countries [8]. To address these information gaps in the UNICEF Europe and Central Asia (ECA) region, we conducted a review and analysis of current anemia prevalence, factors potentially contributing to anemia, and of national policies and programs for reducing anemia. This article summarizes the findings of that review.

The 22 countries in the UNICEF ECA region, categorized by subregions, are: Eastern Europe—Albania, Belarus, Bosnia and Herzegovina, Bulgaria, Croatia, Greece, Kosovo, Moldova, Montenegro, North Macedonia, Romania, Serbia, Türkiye, and Ukraine; The Caucasus—Armenia, Azerbaijan, and Georgia; and Central Asia—Kazakhstan, Kyrgyz Republic, Tajikistan, Turkmenistan, and Uzbekistan.

The review, which was conducted in 2022, aimed to understand which countries in the ECA region had collected representative data on anemia for which population groups since 2010; whether these reported prevalences of anemia had changed since the year 1999; what evidence existed for potential causes of anemia including, but not limited to, iron deficiency; and what policies and programs were in place to address the issue. The priority groups for the analyses were non-pregnant girls and women aged 15–49 y of age (WRA) and preschool-aged children 6–59 mo old (PSC). Information for pregnant (PW) and lactating (LW) women, and other groups was included when available.

## Methods

The review involved 3 stages: examination of the prevalence and trends in anemia and iron deficiency among different population groups through a review of national survey data since 2010; a literature review to explore the evidence for underlying determinants of anemia; and collection and review of relevant program and policy documentation.

All searches focused on a timeframe from 1 January, 2010, to 31 August, 2022. This ensured that the findings were relevant to current nutritional status, interventions, and strategy development.

### Nationally representative survey data

Demographic and Health Surveys (DHS), UNICEF Multiple Indicator Cluster Surveys (MICS), and National Nutrition, Micronutrient, and Nutrition and Health Surveys (NS) were reviewed to establish the most recent estimates of the prevalence of anemia and iron deficiency. The availability of national data from any other surveys was checked through the literature review, communication with UNICEF country office staff, and a review of source data for anemia prevalence in online databases and publications available from the WHO Vitamin and Mineral Nutrition Information System and its regional data base, and the websites of FAO, IFAD, WFP, World Bank, and UNICEF.

Reports of nationally representative anemia and iron deficiency data were examined to determine sample sizes for different population groups, the methods used for blood collection and hemoglobin measurement, whether adjustments were made to hemoglobin for altitude and smoking, or to ferritin measurements for inflammation. National anemia data were examined for any likely associations with age, socioeconomic status, residential and geographic location, and sex, where disaggregation by these factors was reported.

For each country with a national survey report that included anemia since 2010, additional searches were conducted to find any previous national surveys of anemia prevalence (going back to 1999) so that changes in reported anemia prevalence over time could be investigated.

In all cases, anemia was defined by the primary survey analysts and the reported methodology for all national surveys presented used hemoglobin cut-offs in accordance with the 2011 WHO guidelines [9]. We did not have access to the original datasets and therefore used reported prevalences and the same 2011 criteria to categorize anemia prevalence as a public health problem of mild (5%–19.9%), moderate (15%–39.9%), or severe (40% and above) significance.

### Literature search strategy and selection criteria

The literature review focused on information about possible determinants of anemia in the region. Subnational studies, including hospital-based studies, were included where relevant.

Clearly defined search parameters (see search framework in Supplemental Table 1) were employed which respond to the UNICEF regional office framework of diets, practices, and services as both determinants of anemia and avenues for interventions to prevent and control anemia. Key search terms were ∗anemia∗, ∗anemia∗, ∗iron∗ to locate global and regional articles, then ∗anemia AND [country]∗, ∗anemia AND [country], ∗iron AND [country]∗ for each of the 22 countries in the region.

Searches were conducted through PubMed, Medline, and the Cochrane (CENTRAL Register of Controlled Trials and Database of Systematic Reviews). Peer-reviewed publications and grey literature were considered eligible for inclusion. No initial restriction was made by study design or language of publication.

Title and abstract screening, guided by the search framework, was divided among 3 researchers (JK, RB, and TW) to complete independently for different countries. Where abstracts were in a language other than English, an online translation platform was used to examine the contents. If the article was deemed relevant, the full-text article was translated using an online translator or through UNICEF country office support. Full-text analysis and categorization of included papers were conducted by 2 researchers (JK and TW/RB) for each article. All included papers were read in full and summarized, including notes on the appropriateness of study design where applicable. Reference lists of eligible publications were searched for relevant titles. Papers found to be irrelevant to our research question or redundant after full-text reading were excluded, using discussion and consensus where needed.

### Online survey

An online survey was developed for completion by UNICEF country offices. The survey included questions about available data on anemia and iron deficiency; evidence of differences in prevalence by socioeconomic, demographic, or other influences; evidence for potential factors contributing to anemia, for example infant and young child feeding practices, access to iron-fortified foods and micronutrient supplementation, and household access to improved water and sanitation; whether anemia reduction targets existed for any population group and what national policies or programs for interventions aimed at reducing anemia or iron deficiency among different groups were being implemented and how.

### Policy and program review

The online survey, email communication with UNICEF country offices, and online document searches were used to obtain policy and program documentation relevant to addressing anemia, iron deficiency, or other likely underlying causes of anemia in the ECA region. Documentation collected included national nutrition plans, sector policies and strategies, national guidelines, and legislation/regulation documents; as well as relevant regional UNICEF and other UN or European Union policies, program guidelines, and strategic approaches.

## Results

Information on anemia data and related legislation, policies, and programs was received from 15 UNICEF country offices and the literature search delivered >6000 hits. Together these sources resulted in 268 documents of potential relevance to the regional review. An overview of which UNICEF country offices provided responses, the type and number of relevant documents sourced by country, and which countries had national survey reports including anemia data are presented in Supplemental Table 2.

### Nationally representative data for anemia in ECA countries

**FIGURE 1:**
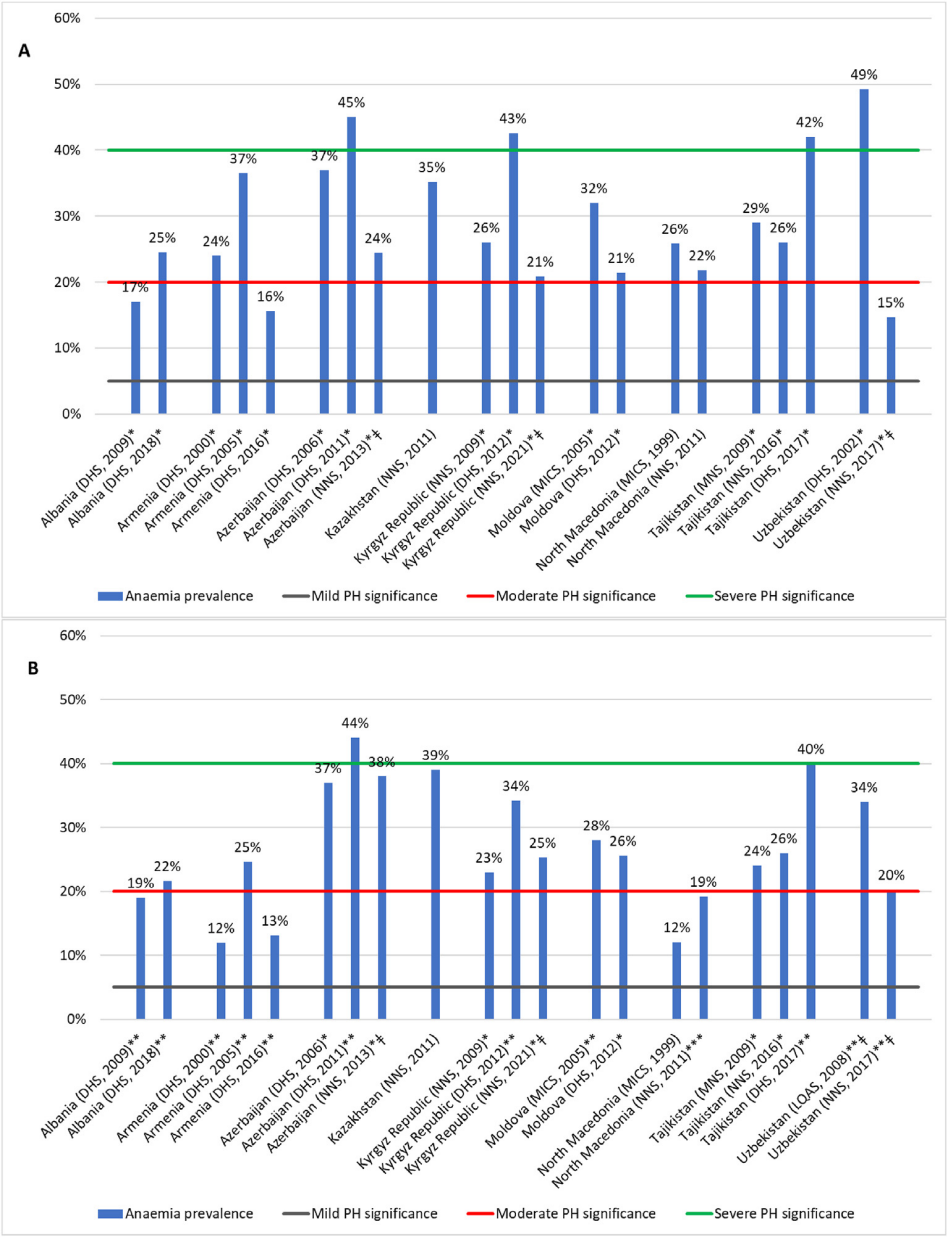
Prevalence of an**e**mia among PSC (A) and WRA (B), latest available data and change over time since 1999, for ECA countries with national survey data. Cut-offs shown for WHO categorization of public health (PH) significance of anemia based on prevalence estimated from blood hemoglobin. ∗Hemoglobin adjusted for altitude. ∗∗Hemoglobin adjusted for altitude and smoking. ∗∗∗Hemoglobin adjusted for smoking (no star indicates that no information on hemoglobin adjustment was available in the report). All prevalence figures and adjustments, WHO 2011 [9]. ^ǂ^Hemoglobin measured in venous blood. All other data shown are based on measurements from capillary blood.

Nine of the 22 countries had anemia prevalence data for PSC and/or WRA from ≥1 of a DHS, MICS, or NS conducted since 2010 (see Table 1 and Figure 1A and B) [[10], [11], [12], [13], [14], [15], [16], [17], [18], [19], [20], [21], [22], [23], [24], [25], [26], [27], [28], [29], [30], [31]]. A further 2 surveys reported nationally representative anemia prevalence among slightly different population groups; hence, they are included in Table 1, but are not included in Figure 1. These were a national anemia survey in Romania in 2010 [33], which was conducted among children 6–23 mo old (anemia prevalence 46%), and a nutrition survey in Türkiye (2017) [34] that reported anemia prevalence among women 15 y and above (22%).

**TABLE 1:** Methodological details for nationally representative surveys of anemia prevalence in the ECA region.

Country	Year of the survey	Type of survey	Population group and sample size^1^ (*n*)	Type of blood collection	Hemoglobin measurement method^1^	Data adjusted for altitude and smoking
Albania	2018 [10]	DHS	PSC (1778) WRA (9440) PW (233) LW (670) Men 15–49 y (4148)	Capillary	HemoCue Model not stated	Yes, both
	2009 [29]	DHS	PSC (1322) WRA (6933) PW (149) LW (361) Men 15–49 y (2897)	Capillary	HemoCue 201+	Yes, both
Armenia	2016 [11]	DHS	PSC (1349) WRA (5261) PW (156) LW (351)	Capillary	HemoCue 301+	Yes, both
	2005 [23]	DHS	PSC (1106) WRA (5609) PW (176) LW (294)	Capillary	HemoCue Model not stated	Yes, both
	2000 [20]	DHS	PSC (1334) WRA (5694) PW (169) LW (274)	Capillary	HemoCue Model not stated	Yes, both
Azerbaijan	2013 [12]	NS	PSC (1111) WRA (2706) PW (167)	Venous PSC and WRA Capillary PW	HemoCue 201+	Yes, altitude
	2011 [24]	DHS	PSC (2107) WRA (8229) PW (337) LW (469)	Capillary	HemoCue Model not stated	Yes, both
	2006 [21]	DHS	PSC (1840) WRA (7392) PW (289) LW (431)	Capillary	HemoCue Model not stated	Yes, altitude
Kazakhstan^2^	2011 [13]	NS	PSC (1338) WRA (1214) PW (89)	Capillary	HemoCue Model not stated	No information provided
Kyrgyz Republic	2021 [14]	NS	PSC (1211) WRA (615) PW (142) LW (212) Children 5–9 y (1401) Adolescent girls 10–18 y (858)	Venous PSC and WRA Capillary PW	HemoCue 301+ CBC on a subset^3^	Yes, altitude
	2012 [25]	DHS	PSC (3971) WRA (6162) PW (540) LW (1300)	Capillary	HemoCue 201+	Yes, both
	2009 [22]	NS	PSC (1743) WRA (1162)	Capillary	HemoCue 301+	Yes, altitude
Moldova	2012 [15]	MICS	PSC (1422) WRA (4802) PW (165) LW (397)	Capillary	HemoCue Model not stated	Yes, both
	2005 [31]	DHS	PSC (1364) WRA (6637) PW (168) LW (333)	Capillary	HemoCue Model not stated	Yes, altitude
North Macedonia	2011 [16]	NS	PSC (1080) WRA (755)	Capillary	HemoCue 301+	Yes, smoking
	1999 [32]	MICS	PSC (1079) WRA (1018)	Capillary	HemoCue Model not stated	No information provided
Romania	2010 [33]	Anemia survey	6–23-mo-olds (1532)	Capillary	HemoCue Model not stated	No information provided
Tajikistan	2017 [17]	DHS	PSC (6036) WRA (7986) PW (792) LW (1859)	Capillary	HemoCue 201+	Yes, both
	2016 [[27], [30]]	NS	PSC (2096) WRA (2125)	Capillary	HemoCue Model not stated	Yes, altitude
	2009 [26]	NS	PSC (2175) WRA (2138)	Capillary	HemoCue Model not stated	Yes, altitude
Türkiye	2017 [34]	NS	Women 15 y+ (6115) Men 15 y+ (5036)	Venous	CBC	No information provided
Uzbekistan	2017 [18]	NS	PSC (1936) WRA (2207) PW (243)	Venous PSC and WRA Capillary PW	HemoCue 301+	Yes, altitude
	2008 [19]	LC-LQAS	WRA (2584)	Venous	HemoCue 301+	Yes, both
	2002 [28]	DHS	PSC (2305)	Capillary	HemoCue 201+	Yes, altitude

Abbreviations: DHS, Demographic and Health Survey; LC-LQAS, Large Country-Lot Quality Assurance; MICS, Multiple Indicator Cluster Survey; NS, Nutrition/Micronutrient/Nutrition and Health Survey; LW, lactating women; PSC, preschool children, 6–59 mo of age; PW, pregnant women; WRA, non-pregnant women of reproductive age, 15–49 y (where LW is indicated as a separate group then WRA are non-lactating women. Where LW are not indicated then WRA probably includes lactating women, however it is not specified in the reports).

Data were weighted to account for the sample design for all surveys, except Kazakhstan and Romania where no information about sample design adjustments was provided.

The initial search was conducted for national surveys since 2010. Where survey reports existed, a search was also conducted for previous surveys (from 1999) in the same countries to provide an indication of the trend in anemia prevalence, information for all surveys is included.

*n* = weighted sample size except for Azerbaijan 2013, Kyrgyz Republic 2009 and 2021, Turkey 2017, and Uzbekistan 2008 and 2017, which are un-weighted numbers, and North Macedonia 1999 where this is not specified in the report.

1 HemoCue AB, Ängelholm, Sweden. CBC = complete blood count – no information was provided about the equipment used.

2 The 2011 Kazakhstan survey report refers to a 2008 national survey that included anemia; however, it was not possible to find sufficient information to determine any details about the methodology, therefore the data are not included in this review.

3 The survey report notes that the mean hemoglobin concentration measured by the CBC was 4g/L (∼3%) lower than the concentration obtained by the HemoCue 301+ across all population groups.

Figure 1A and B shows the most recent nationally representative estimates for anemia since 2010 for PSC and WRA, respectively, with an indication of public health significance [9]. The figure also includes prevalence data from previous surveys in these countries, where available since 1999 (8 countries), to indicate the reported trend in anemia prevalence over time.

Table 1 indicates which surveys included nationally representative anemia prevalence data for PW (8 countries), LW (5), adolescent girls (10–19 y old) (1), and children 5–9 y old (1). The table also provides details on survey sample size and methods used for blood collection, hemoglobin assessment, and data weighting and adjustment. All surveys collected capillary blood except for the surveys in Azerbaijan (2013) [12], the Kyrgyz Republic (2021) [14], Türkiye (2017) [34], and Uzbekistan (2008, 2017) [18,19] where venous blood was collected for PSC and WRA. Hemoglobin was analyzed using a HemoCue (HemoCue AB) in all surveys except for in Türkiye (2017) and for a subset of samples in the Kyrgyz Republic (2021) where automated analyzer complete blood counts were also conducted. It is now understood that methods of blood collection and analysis can affect hemoglobin results. Results in this article are presented as reported; however, the potential influence of the different methodologies and instrumentation used is included in the discussion.

The most recent prevalence figures indicate that anemia was a problem of severe public health significance among both PSC and WRA in Tajikistan (2017) [17], a public health problem of moderate significance among PSC in 6 countries (Albania (2018) [10], Azerbaijan (2013) [12], Kazakhstan (2011) [13], the Kyrgyz Republic (2021) [14], Moldova (2012) [15], and North Macedonia (2011) [16]) and among WRA in 6 countries (Albania (2018) [10], Azerbaijan (2013) [12], Kazakhstan (2011) [13], Kyrgyz Republic (2021) [14], Moldova (2012) [15], and Uzbekistan (2017) [18]), and a mild public health problem in Armenia (2016) [11] (PSC and WRA), in North Macedonia (2011) [16] (WRA), and in Uzbekistan (2017) [18] (PSC).

Figure 1 shows inconsistent results for changes in anemia prevalence over time for the 8 countries with ≥2 sets of national data since 1999. Three survey reports were available since 1999 for 4 countries and in these, the reported anemia prevalence in the first surveys for both PSC and WRA in Armenia in 2000 [20], Azerbaijan in 2006 [24], and the Kyrgyz Republic in 2009 [22] was lower than in the subsequent survey (in 2005 [23], 2011 [24], and 2012 [25], respectively); however, a similar prevalence to the first survey was reported in the latest survey for PSC and WRA from Armenia (2016) [11] and the Kyrgyz Republic (2021) [14], and for WRA from Azerbaijan (2013) [12]. The most recently reported prevalence of anemia among PSC in Azerbaijan (2013) indicated a decrease from both 2006 [21] and 2011 [24]. A high absolute change in anemia prevalence over time was reported for Tajikistan, from 29% (PSC) and 24% (WRA) in 2009 [26] to 42% and 40%, respectively, in 2017 [17]. However, a national micronutrient survey in Tajikistan 2016 [30] found a lower anemia prevalence of 26% among both PSC and WRA, making the situation unclear.

In the 4 countries with 2 survey reports since 1999, the reported prevalence of anemia decreased between 2005 and 2012 in Moldova (PSC) [15,31], and from 2002 to 2017 (PSC) and 2008 to 2017 (WRA) in Uzbekistan [18,19,28]. An increased anemia prevalence among PSC was found from 2009 to 2018 in Albania [10,29] and there was little change between 1999 and 2011 in North Macedonia [16,32]. For WRA over the same periods of time, an increase in anemia prevalence was found in North Macedonia [16,32] and little change in prevalence was found in Albania [10,29], and Moldova [15,31].

In the 8 and 5 countries with national anemia data for PW and LW since 2010, respectively, PW tended to have a slightly higher prevalence of anemia than non-pregnant women. See Table 2 for the reported anemia prevalence among PW, LW, and other population groups with data [[10], [11], [12], [13], [14], [15], [17], [18]]. The significance of the problem of anemia for PW in terms of WHO public health categorization was the same as for non-pregnant women for 5 countries; however, in Azerbaijan (2013), Kazakhstan (2011), and the Kyrgyz Republic (2021), the public health significance of anemia was moderate for WRA, but severe for PW.

**TABLE 2:** Prevalence of anemia among pregnant and lactating women and other population groups from national surveys in the ECA region since 2010.

	Anemia prevalence (%)		
Country (year of survey)	Pregnant women	Lactating women	Other population groups
Albania (2018) [10]	25.5%	22.2%	Men 15–49 y 11.5%
Armenia (2016) [11]	11.2%	16.1%	
Azerbaijan (2013) [12]	40.4%		
Kazakhstan (2011) [13]	43.8%		
Kyrgyz Republic (2021) [14]	49.3%	39.2%	Adolescent girls 10–18 y 14.6%^1^
			Children 5–9 y 7.8%
Moldova (2012) [15]	26.3%	27.7%	
Tajikistan (2017) [17]	42.1%	46.1%	
Uzbekistan (2017) [18]	32.8%		

1 A prevalence of 6% among premenarche girls (*n* = 361) and 21% among postmenarche girls (*n* = 497).

### Factors potentially associated with anemia in the ECA region

#### Demographic and socioeconomic factors

**FIGURE 2:**
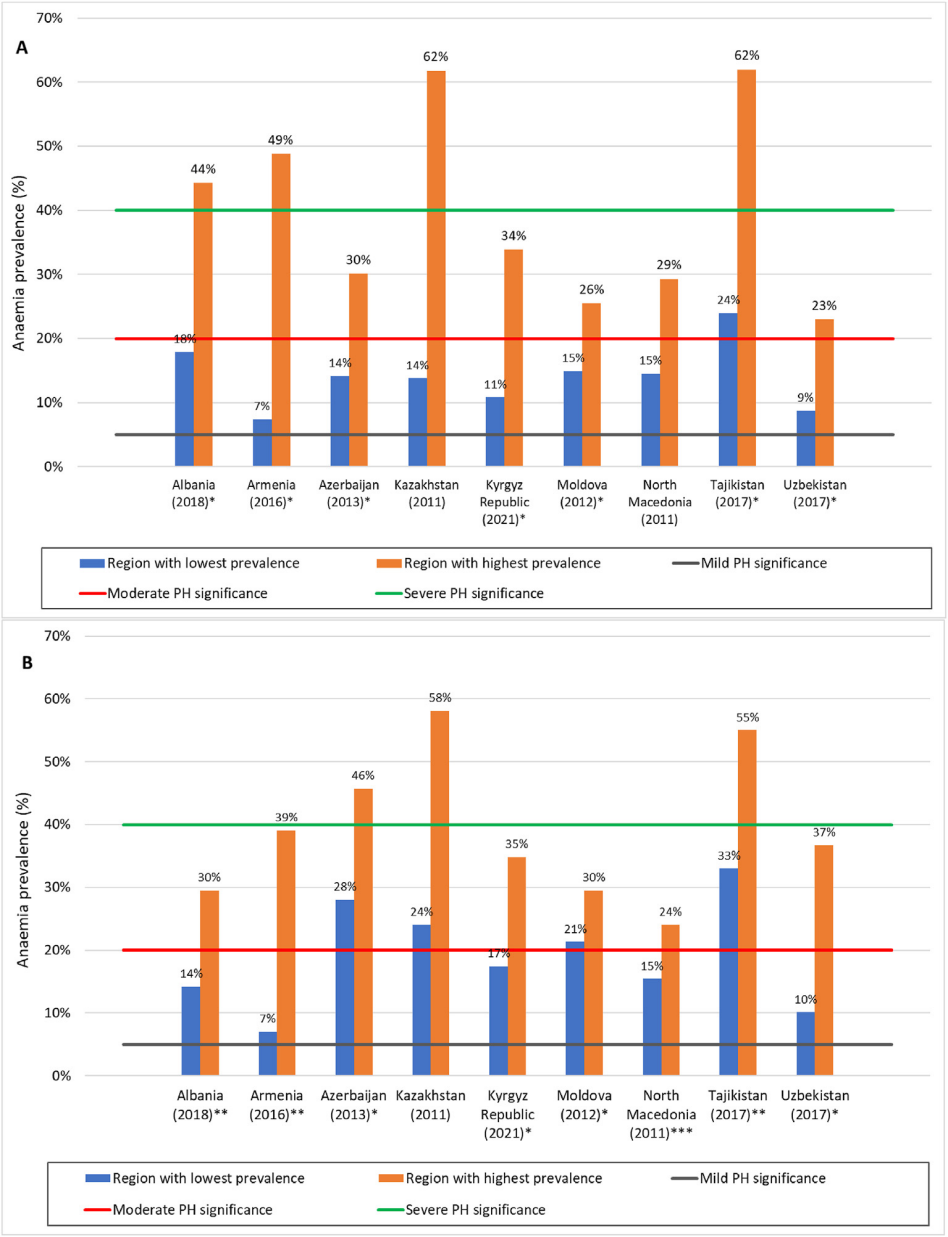
Range in the prevalence of an**e**mia among PSC (A) and WRA (B) for the geographical region of each country with lowest prevalence compared with the region with highest prevalence, for ECA countries with data from a national survey since 2010. Cut-offs shown for WHO categorization of public health (PH) significance of anemia based on prevalence estimated from blood hemoglobin. ∗Hemoglobin adjusted for altitude. ∗∗Hemoglobin adjusted for altitude and smoking. No star indicates that no information on hemoglobin adjustments was available to the report authors. All prevalence figures and adjustments, WHO 2011 [9].

Large subnational regional differences (≤5 times) in anemia prevalence among PSC and WRA were reported within countries with national survey reports. Figures 2A (PSC) and 2B (WRA) show anemia prevalence, along with categorization of anemia in terms of a public health problem, in the subnational regions with the lowest and highest levels. The names of these regions with lowest and highest prevalence for PSC and for WRA are provided in Supplemental Table 3.

All 8 of the most recent national surveys showed a consistently higher prevalence of anemia among 6–23-mo-olds than among 24–59-mo-olds, with ≤5 times higher prevalence among 6–11-mo-olds when compared with children 48–59 mo of age. There was no clear trend with age among WRA. The Kyrgyz Republic is the only country in the region with recent national anemia data for adolescent girls 10–19 y of age where it was reported that ∼15% of adolescent girls were anemic. The prevalence among this group increased with age (8% among 10–12-y-olds and 24% among 16–18-y-olds) and was significantly higher in menarche (21%) than in premenarche (6%) girls [14]. Among PW, subnational studies of anemia in 4 countries (Greece [35], North Macedonia [36], Romania [37], and Türkiye [38]) reported a significantly higher prevalence of anemia among pregnant adolescents <19 y of age, when compared with the prevalence among PW aged 20–24 (North Macedonia and Romania) or 20–34 (Greece and Türkiye) years.

Six of the most recent national surveys [Albania (2018), Moldova (2012), North Macedonia (2011), Armenia (2016), Kyrgyz Republic (2021) and Tajikistan (2017)] reported a notably higher prevalence of anemia among PSC associated with rural residence, lower household wealth quintile (except Armenia), and lower maternal education (except Tajikistan). Aside from in the Kyrgyz Republic, the statistical significance of these results was not tested or presented. Statistical analyses reported for nationally representative data for anemia among 6–23-mo-olds in Romania [33] described significant associations with anemia for these same 3 demographic factors. An apparent association between higher anemia among PSC with lower maternal education was also observed in the survey reports from Azerbaijan (2013) and Kazakhstan (2011). The reported prevalence of anemia among WRA showed little apparent difference by residence type, except in Moldova (2012) where it appeared higher among rural residents, and Azerbaijan (2013) where it was reported as higher among urban residents. However, reports indicated a higher anemia prevalence among WRA with lower household wealth quintile in Albania (2018), Moldova (2012), North Macedonia (2011) and Tajikistan (2017); with lower maternal education in Albania (2018), Armenia (2016), Kazakhstan (2011), Moldova (2012), North Macedonia (2011), and Tajikistan (2017); and with an increased number of pregnancies in Albania (2018), Moldova (2012), Kazakhstan (2011), and Tajikistan (2017).

### Iron and other micronutrient deficiencies

Five of the national surveys conducted since 2010 included an assessment of iron deficiency as well as anemia for WRA and PSC. Table 3 presents the findings for the prevalence of iron deficiency in these 5 countries along with the indicator used to determine iron deficiency, the cut-off value used, and whether adjustments were made to account for inflammation [[12], [13], [14],^18^,[27], [39],^40^].

**TABLE 3:** Prevalence of iron deficiency among PSC and WRA from national surveys in the ECA region since 2010.

Country (year of survey)	Iron deficiency prevalence (%)		Indicator of iron deficiency
	PSC	WRA	
Azerbaijan (2013) [12]	15.0% *N* = 185	34.1% *N* = 930	Plasma ferritin^1^ <12 μg/L (PSC) <15.0 μg/L (WRA) or sTfR >8.3mq/L (PSC and WRA)
Kazakhstan (2011) [13]	38.1% *N* = 1083	43.8% *N* = 471	Plasma ferritin^2^ <12 μg/L (PSC) <15.0 μg/L (WRA)
Kyrgyz Republic (2021) [14]	47.0% *N* = 1161	55.9% *N* = 1149	Plasma ferritin^3^ <12 μg/L (PSC) <15.0 μg/L (WRA)
Tajikistan (2016) [30]	52.4% *n* = 2005	34.5% *n* = 2023	Serum ferritin^1^ <15 μg/L (PSC) <12 μg/L (WRA) or Transferrin receptor >3.3 μg/mL (PSC and WRA)
Uzbekistan (2017) [18]	54.7% *N* = 1736	48.1% *N* = 2077	Serum ferritin^3^ <12 μg/L (PSC) <15.0 μg/L (WRA)

Abbreviation: sTfR, soluble transferrin receptor.

1 Ferritin adjusted for inflammation based on elevated CRP and AGP [39].

2 Iron deficiency based on ferritin was only assessed for samples with serum CRP <5 mg/L.

3 Ferritin adjusted for inflammation based on a correction algorithm [40].

A published analyses using multivariable Poisson regression models to determine risk factors for anemia using national survey data for Azerbaijan [41] found that the population attributable fractions for anemia among PSC and WRA were 17.6% and 43.2%, respectively, for iron deficiency, 13.6% for inflammation and 3.4% for recent lower respiratory infection (PSC only for both factors), and 3.7% for vitamin A insufficiency (only assessed for WRA). A similar article applying a multivariable Poisson regression model to data from the 2017 national survey in Uzbekistan reported the relative risk for anemia among WRA was 4.7 for iron deficiency and 1.5 for vitamin A insufficiency [19].

Other significant associations reported from nationally representative data for PSC include findings of higher anemia with low dietary diversity (<5 food groups) in North Macedonia (2011), and with belonging to a minority ethnic group in North Macedonia (2011) and in Romania (2010, 6–23-mo-olds).

### Policy and programming landscape

Information provided by the 15 UNICEF country offices along with supplementary material from internet searches for these countries revealed that all 15 countries have ≥1 policy, program, or strategy pertaining to anemia prevention and control targeting at minimum 1 population subgroup. The countries that reported having, or recently having had, specific targets for anemia reduction were Albania, Armenia, Bosnia and Herzegovina, the Kyrgyz Republic, Tajikistan, Türkiye, and Uzbekistan.

The strategies for anemia prevention and control reported for the 15 countries focused mainly on prevention of iron deficiency and included iron supplementation for pregnant women, counseling on appropriate complementary feeding to support dietary diversity and iron absorption, and iron fortification of wheat flour. An overview of policies and programs reported, or otherwise found, for the 15 countries is shown in Table 4. All 5 Central Asian countries, Armenia, Bosnia and Herzegovina, Moldova, and Türkiye had implementation documents for ≥6 interventions. It was noted that Uzbekistan reported the highest number of interventions and national data from 2017 indicated that it was also the country with the largest absolute reduction in anemia in the period from 2002 for PSC and 2008 for WRA.

**TABLE 4:** Policies and programs for prevention of anemia and iron deficiency in countries of the ECA region, as reported in 2022.

Subregion and country	Wheat flour fortification	Screening and treatment for anemia in pregnancy	Routine iron supplementation	MNP for 6–23-mo-olds	Promotion of appropriate complementary feeding	EBF promotion	Delayed cord clamping	Dietary counseling for anemia prevention (ANC)	De-worming
Caucasus									
Armenia	No	National	PW	No	National	National		National	No
Azerbaijan	No	National	PW, LW, PSC	No	No	National	National	National	
Central Asia									
Kazakhstan	Mandatory	National	PW, LW	No	National	National	No	National	No
Kyrgyz Republic	Mandatory	National	PW, LW, ADG	No, but included in an action plan.	National	National	National	National	National
Tajikistan	Mandatory	Subnational	PW (subnational)	No, but included in an action plan.	Subnational	National	No	National	Subnational
Turkmenistan	Mandatory	National	PW	No	National	National	No	National	National
Uzbekistan	Mandatory	National	PW, LW, ADG	National	National	National	National	National	National
Central and Eastern Europe									
Bosnia and Herzegovina	No	National	PW	Subnational	National	National		National	No
Croatia	No		PW^1^		National	National	National	National	
Moldova	Mandatory	National	PW^1^, LW^1^		National	National		National	
Montenegro	No	National^1^	PW^1^, LW^1^	No	National	National			
North Macedonia	No	No	No	No		National			
Serbia	No	National	PW	No	National	National	No	National	No
Türkiye	No	National	PW^1^	No	National	National	National	National	No
Ukraine	No	No	No	No	No	No	No	No	No

Abbreviations: ADG, adolescent girl; ANC, as part of antenatal care; EBF, exclusive breastfeeding; LW, lactating woman; MNP, micronutrient powder; PSC, preschool children; PW, pregnant woman.

Blank cells = no information available.

1 Information was reported by UNICEF, however no specific policy or program documents were provided.

We investigated evidence for implementation of anemia prevention activities; however, it was difficult to obtain a comprehensive picture for this. Our conclusion based on available information and follow-up with UNICEF country offices was that most countries lacked a coherent, multisectoral, approach with strong monitoring and evaluation, when compared with WHO recommendations [1]. For example, anemia and iron deficiency-related indicators appeared to be missing from health and nutrition monitoring mechanisms for the targeted age groups.

Although iron supplementation of PW or WRA was implemented in 13 countries, it was apparent from the documentation available that most protocols did not adhere to WHO guidelines, and/or did not contain complete information on the supplementation dose or schedule. Fortification strategies, laws, or programs for iron fortification of wheat flour exist in 6 of the 15 countries; however, enforcement and monitoring protocols were often weak. Fortification was only implemented at scale (∼90% coverage) in Turkmenistan [42], whereas estimates were ∼40%–50% for Kazakhstan [43] and Uzbekistan [18], ∼25% in the Kyrgyz Republic [14], and <10% in Tajikistan [44] and Moldova [45].

Despite strong WHO global guidance for prevention of anemia, we found no specific UN, EU, or other, regional policies or guidance for prevention or control of anemia.

## Discussion

This article provides a valuable update on the prevalence of anemia in the ECA region and associated prevention and control policies and programs in place. Nationally representative data for anemia in ≥1 population group were found for around half of the countries in the region and for iron deficiency in less than a quarter.

The authors recognize that results, based on cut-offs for anemia defined in the 2011 WHO guidelines [9], show a prevalence of anemia among 6–23-mo olds that is higher than it would be using the revised (2024) WHO guidelines [46]. Applying the 2024 WHO recommendations would also result in small changes to the reported prevalence across all groups due to amendments to the recommended adjustments for smoking and altitude, and for PW where a different cut-off for anemia has now been established for the second trimester.

Equally important are the implications of the blood collection and measurement methods on reported anemia prevalence in the national surveys. The 2024 WHO guidelines recommend the “Use of venous blood, automated hematology analyzers and high-quality control measures” for the assessment of hemoglobin concentration in populations, which was the methodology used in only 2 surveys (a subsample in the Kyrgyz Republic 2021, and Türkiye 2017) in this review. The high measurement variability for hemoglobin from capillary blood, especially single drop capillary specimens, as used in most of the surveys referenced in this article is now well understood [47]. In addition, without prior adjustment for device bias, different HemoCue models produce results that are not considered robust enough to make reliable comparisons between surveys conducted by different teams over many years [46,47]. These methodological issues may help explain some of the unusual differences in anemia prevalence observed over a short period of time, for example from 2011 to 2013 in Azerbaijan and from 2016 to 2017 in Tajikistan. However, we are confident that our review remains important in underlining that anemia remains a significant public health problem in all countries where data were available. The review also indicates that, with the exception of Uzbekistan, no country in the region is on track to achieve the World Health Assembly 2025 target of a 50% reduction in anemia in WRA from 2012 levels.

The large and important differences in anemia prevalence within each country show that anemia is a more serious public health concern than indicated by the national prevalence data for some more vulnerable groups. These discrepancies require more in-depth, systematic, subnational investigations across the region to improve the existing evidence base for nutritional and non-nutritional causes of anemia and to enable the design and implementation of coherent national policy frameworks to effectively address the problem, as highlighted in recent papers [4,48,49].

Information from the national survey reports and related publications referenced in this article, together with current global understanding of causal factors for anemia [6,7], suggests that iron deficiency is a major contributing factor to anemia among WRA in the region. Other contributing factors suggested by available evidence are socioeconomic status (as reported above); the persistence of suboptimal infant and young child feeding practices among some groups in the region [50], particularly low achievement of a minimum acceptable diet among Roma children aged 6–23 mo [[51], [52], [53]]; deficiencies of folate and vitamin A [14,41]; a high adolescent birth rate among some ethnic minority groups or otherwise vulnerable, groups [[51], [52], [53], [54]]; and infection with soil-transmitted helminths, especially in the Central Asian Republics [55].

On the basis of documents available to us, there are potential program and information gaps that could be addressed regarding iron supplementation for non-pregnant WRA and PSC, protocols and training for delayed cord clamping, assessment and treatment of intestinal parasites, and access to improved water, hygiene and sanitation. A lack of monitoring and evaluation data for existing programs limits the ability to ensure that the most vulnerable groups are identified and reached with effective anemia control interventions. On the positive side, reported access to WHO-recommended antenatal care practices [56] and to basic water and sanitation services [57] in the region is generally good, although both appear to be associated with socioeconomic status.

The UNICEF regional office has previously estimated that an investment of $600 million is needed in the region to meet the World Health Assembly anemia target among WRA and made a compelling economic argument that each dollar invested in the package of prevention interventions would yield ∼$12 in economic returns [58]. The outcome of this review may help determine how and where to make such an investment if it becomes possible; however, there remains a need for improved assessment and understanding of anemia etiology in the ECA region.

### Limitations and strengths of the review

The review relied on information the authors could find online or that was provided by UNICEF country offices. As such, program-related information was lacking for 7 countries in the region.

As discussed above, there are known limitations to the accuracy and reliability of the hemoglobin data and interpretation of anemia prevalence in the source survey reports for this review, which were based on 2011 WHO guidance.

A strength of the review was the time allocated to searching the literature and communicating with UNICEF country offices to verify and supplement any information received or found.

### Conclusions and recommendations

Anemia remains a significant public health and development issue across the ECA region. It is now important to engage with nutrition-specific and nutrition-sensitive sectors, including agriculture, health, education, WASH, and social protection, to strengthen available data on the subnational prevalence and etiology of anemia and use this to develop effective and coordinated plans to establish and achieve national anemia reduction targets for WRA and PSC.

## Author contributions

The authors’ responsibilities were as follows – AY, TW, RB, JK: designed the study; JK: wrote the paper; TW, RB, JK: prepared the final content; and all authors: read and approved the final version of this manuscript.

## Funding

UNICEF utilized global thematic funds for nutrition to support this regional analysis as part of the regional priorities for nutrition in UNICEF Europe and Central Asia Regional Office (ECARO). NutritionWorks was commissioned to conduct the work by UNICEF ECARO who were involved with the overarching design of the work, but were not involved with implementation, analysis, and interpretation beyond facilitating communication with UNICEF country offices.

## Conflict of interest

The authors report no conflicts of interest.
