# Supplementary material for: A Review of Anemia Prevalence, and Prevention and Control Strategies, in the Eastern Europe and Central Asia Region
Source: Curr Dev Nutr. 2024 Oct 15;8(12):104477. doi: 10.1016/j.cdnut.2024.104477 (PMC11663958; doi:10.1016/j.cdnut.2024.104477)
Supplement: multimedia component 1 [file mmc1.docx]

## **A review of anaemia prevalence, and prevention and control strategies, in the Eastern Europe and Central Asia region** – J Knowles

### Supplementary Table 1: Framework for the literature search strategy

**Outcome**: Prevalence of anaemia – identifying iron deficiency anaemia where possible

| **Population** | **Factors – diets** | **Factors – practices** | **Factors – services** |
| --- | --- | --- | --- |
| **Girls and women of reproductive age** (15 to 49 years), non-pregnant, disaggregating data specific to adolescent girls wherever possible  **Pregnant women,** disaggregating data on adolescent girls where possible  **Children under 5 years**  **Children 6 to 15 years***  ***Living in the three clusters of countries of Central and Eastern Europe, the Caucasus and Central Asia, as defined by UNICEF*** | Traditional diets that promote/inhibit iron absorption, e.g. high meat consumption and consumption of high-phytate foods such as cereals, legumes and nuts  Food taboos in pregnancy  WRA and children achieving minimum recommended dietary diversity  Accessibility of fortified foods  Children, adolescents or WRA presenting as underweight or overweight | IYCF practices  Dietary practices and household care of pregnant women  Adolescent dietary habits and food consumption  Intra-household food-sharing practices  Consumption of fortified foods  Use of iron and other micronutrient supplements (including point-of-use fortification powders, etc.)  Use of ANC, delivery and postpartum care by pregnant women/new mothers  Use of iron supplements for the first three months postpartum  Uptake of childhood vaccinations (in particular rotavirus)  Appropriate care seeking and treatment for childhood diseases  WASH practices  Household decision making and food budget practices  Family planning, early marriage, attitudes on contraception, birth spacing | Iron fortification and flour fortification legislation and implementation  *+ other micronutrient (e.g. vitamin A /B vitamins) fortification*  Iron supplementation  *+ other micronutrient (vitamin A /B vitamins) supplementation (including point-of-use powders, etc.*  HIV/tuberculosis treatment and prevention  Helminth treatment/deworming  Access to (quality) ANC, delivery and postpartum care  Assessment and treatment of anaemia as part of ANC  Education for pregnancy and IYCF  Routine delayed cord clamping  Iron supplementation for the first three months postpartum  Access to vaccination  Access to appropriate treatment for childhood diseases  School (primary or secondary) meal food provision and minimum standards; health and nutrition education  Family planning services  *All the above services will be examined in terms of equitable provision and access, as far as the literature allows, e.g. cost of health services, location and socioeconomic factors related to attendance. Analyses will also consider differentiation of prevalence and interventions by sex in school-age children and adolescents.* |

*For children aged 6 to 15 years, the review primarily relied on information in Mates E, Lelijveld N, Ali Z, Sadler K, Yarparvar A, Walters T, et al. Nutrition of School-Aged Children and Adolescents in Europe and Central Asia Region: A Literature and Survey Review. Food Nutr Bull. 2023 Apr 5;037957212311630.

### Supplementary Table 2. Summary of the type and number of documents included in the review

Indication of the UNICEF country offices that provided information for the regional review, and the number of documents (referring to surveys, studies and analyses since 2010, and to current legislation regardless of the year of enactment) that were included in the final literature review matrix. These were found through online literature searches or provided by UNICEF country office personnel.

| **Country** | **UNICEF offices responding to information requests** | **Documents included in the literature review*** | | | |
| --- | --- | --- | --- | --- | --- |
|  |  | **Policy and programme documents, including legislation and action plans** | **Situation analyses and reports from relevant surveys (sub-national or not including anaemia)** | **Research studies** | **National survey of anaemia since 2010** |
| **TOTAL** | **15** | **78** | **76** | **118** |  |
| **Albania** |  | 2 | 5 | 10 | ✓ |
| **Armenia** | ✓ | 11 | 3 | 1 | ✓ |
| **Azerbaijan** | ✓ | 4 | 5 | 4 | ✓ |
| **Belarus** |  | 1 | 1 | 1 |  |
| **Bosnia and Herzegovina** | ✓ | 2 | 3 | 1 |  |
| **Bulgaria** |  | 0 | 3 | 0 |  |
| **Croatia** | ✓ | 0 | 5 | 1 |  |
| **Georgia** |  | 5 | 8 | 4 |  |
| **Greece** |  | 0 | 6 | 15 |  |
| **Kazakhstan** | ✓ | 0 | 5 | 4 | ✓ |
| **Kosovo (UNSCR 1244)** |  |  |  |  |  |
| **Kyrgyz Republic** | ✓ | 17 | 6 | 5 | ✓ |
| **Moldova** | ✓ | 4 | 1 | 0 | ✓ |
| **Montenegro** | ✓ | 3 | 2 | 0 |  |
| **North Macedonia** | ✓ | 7 | 2 | 3 | ✓ |
| **Romania** |  | 2 | 6 | 6 | ✓ (6–24-month-olds) |
| **Serbia** | ✓ | 0 | 1 | 1 |  |
| **Tajikistan** | ✓ | 6 | 2 | 3 | ✓ |
| **Türkiye** | ✓ | 6 | 7 | 52 | ✓ (Women 15 years +) |
| **Turkmenistan** | ✓ | 1 | 1 | 0 |  |
| **Ukraine** | ✓ | 6 | 2 | 4 |  |
| **Uzbekistan** | ✓ | 1 | 2 | 3 | ✓ |

**Two situation analyses and two research documents included information from more than one country. These are included in the numbers for each country referenced in the documents.*

*WRA = non-pregnant girls and women of reproductive age, 15 to 49 years*

### Supplementary Table 3. Regions with lowest and highest prevalence of anaemia among pre-school children (PSC) and non-pregnant girls and women aged 15 to 49 years (WRA), by country (associated with Figure 2).

|  | **Lowest prevalence region PSC** | **Highest prevalence region PSC** | **Lowest prevalence region WRA** | **Highest prevalence region WRA** |
| --- | --- | --- | --- | --- |
| **Albania** | Tirana | Dibër | Korçë | Gjirokastër |
| **Armenia** | Armavir | Gegharkuni | Armavir | Gegharkuni |
| **Azerbaijan** | Baku | Aran | Ganja-Gazakh | Baku |
| **Kazakhstan** | Almaty | Mangistau | Almaty | Kzyl-Orda |
| **Kyrgyz Republic** | Bishkek City | Issyk Kul | Osh City | Issyk Kul |
| **Moldova** | Chișinău | South | Chișinău | Centre |
| **North Macedonia** | Pelagoniski | Poloski | Pelagoniski | East |
| **Tajikistan** | Dushanbe | Gorno-Badakhshan Autonomous Oblast | Districts of Republican Subordination | Gorno-Badakhshan Autonomous Oblast |
| **Uzbekistan** | Namangan | Bukhara | Namangan | Tashkent |

### *Full references for the survey sources of these data are available in the main paper.*
